# Integrative approach involving RNA-Seq, foliar traits and growth measurements revealed genotype-specific plasticity on *Eucalyptus* subjected to seasonal water shortage

**DOI:** 10.1186/1753-6561-5-S7-O28

**Published:** 2011-09-13

**Authors:** Emilie Villar, Christophe Plomion, Jean-Marc Gion

**Affiliations:** 1 CIRAD, France; 2 INRA, France

## Background

In the context of climate change, water availability will become an important limiting factor threatening biomass production, particularly in intensively managed forest tree plantations. The complexity of drought stress response calls for integrative approaches combining physiological, anatomical and molecular investigations at different scales ranging from particular cells to the whole plant. Such a strategy was developed for two eucalyptus clones used in industrial plantations in the Republic of Congo and known for their differences in term of productivity and water-use efficiency.

## Methods

The two genetic units used in this study consisted in one more productive hybrid of *E. urophylla* * *E. grandis* (18-50), and one less productive hybrid obtained from open-pollination of E. *alba* (1-41). These genotypes were subjected to two watering regimes (irrigated during two dry seasons, versus non-irrigated) in open field conditions. Growth parameters such height, diameter at breast height, stem, leaf and root biomasses as well as physiological parameters, such carbon discriminationand specific leaf area, were regularly measured over two growing seasons. At the end of the second dry season (i.e. 14 months-old trees), polyphenols accumulation and anatomical measurements were performed on mature leaves. RNA of shoot apices from trees representing the four conditions (i.e. 2 genotypes subjected to 2 watering treatments) were extracted and used to create non-normalized libraries. Using 454/Roche sequencing, we performed digital expression analysis to highlight differentially expressed transcripts showing genotype, treatments and/or genotype by treatment interaction effects. Expression of candidates genes revealed by this first analysis were assessed by RT-qPCR on shoot apices and on mature leaves.

## Results and discussion

Growth parameters and stem biomass analysis revealed that the two studied genotypes presented similar degrees of phenotypic plasticity in response to watering treatment during the dry season. Nevertheless, comparison of growth increments during wet seasons suggested that the more productive genotype had higher ability to recover from water shortage during the dry seasons and to use favorable conditions to increase its above-ground biomass. We found that the more productive genotype displayed greater plasticity of foliar traits in the non-irrigated treatment during the dry season, suggesting that it could adjust more quickly its leaf parameters when rainfalls resume.

On mature leaves from trees subjected to water deficit, we identified mechanisms established to limit water losses by cuticle reinforcement and stomatal density decrease, to preserve leaf turgor by increase of collenchyma layer, to enhance hydraulic conductance with xylem vessel diameter decrease, and to preserve cells from reactive oxygen species through accumulation of hydroxycinnamates. Those adjustments were more intense for the more productive genotype, suggesting that this genotype may entailed higher cost strategies to preserve its leaf functions.

On shoot apices, 454-sequencing provided a catalogue of 129,993 unigenes (49,748 contigs and 80,245 singletons) from the initial sequencing of 398Mb corresponding to 1.14Mreads. Digital expression analysis identified 1,280 contigs displaying differential expression between the two genotypes, 155 contigs between treatments, and 274 contigs displaying genotype x treatment interaction effects.The more productive genotype mainly under-expressed genes related to photosynthetic activity during the dry season, activating genes to reallocate resources through major changes in primary metabolism, whereas the less productive genotype displayed lower levels of variation in gene abundance between treatments. This analysis allowed to reveal a set of candidate genes that could be related to phenotypic variation observed in mature leaves.

## Conclusions

The productivity of the two genotypes used in this study differed essentially in terms of ability to recover from water shortage. These differences could be explained by different strategies of investment in preservation of leaves. In the environmental gradient tested, it seemed that higher investments enabled genotype 18-50 to increase its leaf resistance, and subsequently to recover quickly high growth performance once environmental conditions become favorable. Genes that potentially underlie such physiological plasticity were revealed through integrative approaches. More investigations are now needed to valid their putative roles in phenotypic plasticity in response to water deficit. Forward genetic approaches could be used to screen the variability of gene expression and other more-integrated phenotypic traits, the nucleotide diversity of these candidate genes and their covariations on a larger genetic background.

